# Speech fluency features in distinguishing the cognitively impaired from unimpaired in Chinese‐speaking patients

**DOI:** 10.1002/alz70856_104138

**Published:** 2025-12-26

**Authors:** Yung‐Shuan Lin, Ya‐Lun Cheng, Chih‐Ting Chang, Jong‐Ling Fuh

**Affiliations:** ^1^ Taipei Veterans General Hospital, Taipei, Taiwan; ^2^ National Taipei University of Nursing and Health Sciences, Taipei, Taiwan

## Abstract

**Background:**

Dementia is a degenerative disease affecting memory and speech fluency. Early detection of cognitive decline is vital but often relies on invasive, time‐consuming, and costly methods. Speech fluency analysis may provide a non‐invasive, cost‐effective alternative for detecting early cognitive impairment. This study aimed to identify speech fluency features distinguishing cognitively unimpaired (CU) from cognitively impaired (CI) individuals, with the potential for self‐administered screening tools.

**Method:**

Chinese‐speaking participants from a neurology clinic completed neuropsychological tests (e.g., MMSE, MoCA, BNT, VF) and audio‐recorded passage reading (142 syllables) and picture description tasks. Acoustic features—including number of syllables (NS), pauses (NP), duration (DT), phonation time (PT), speech rate (SR), and average syllable duration (ASD)—were analyzed using Praat. Statistical analyses included ANOVA, logistic regression, and ROC.

**Result:**

The study included 50 CU and 55 CI (39 MCI, 16 dementia) participants. The CI group was older (mean age 73.0 vs. 70.2, *p* =  0.007) and performed worse on cognitive tests (MMSE, MoCA, BNT, VF, DB; all *p* < 0.05). NP and SR were independently associated with CI in both tasks (passage reading: *p* =  0.004 for NP, *p* =  0.037 for SR; picture description: *p* =  0.026 for NP, *p* =  0.042 for SR). DT in passage reading (*p* = 0.016) was also significant. After adjusting for age, sex, and education, NP (*p* = 0.011) and DT (*p* = 0.023) remained significant. A logistic regression model combining NP from both tasks showed the best performance (AUC = 0.746).

**Conclusion:**

Speech fluency features, especially NP, were associated with cognitive impairment. These findings support the potential of speech fluency analysis as a non‐invasive, cost‐effective screening tool for early detection of dementia.

